# Open-Celled Foams from Polyethersulfone/Poly(Ethylene Glycol) Blends Using Foam Extrusion

**DOI:** 10.3390/polym15010118

**Published:** 2022-12-27

**Authors:** Aniket Raje, Prokopios Georgopanos, Joachim Koll, Jelena Lillepärg, Ulrich A. Handge, Volker Abetz

**Affiliations:** 1 Helmholtz-Zentrum Hereon, Institute of Membrane Research, Max-Planck-Strasse 1, 21502 Geesthacht, Germany; 2 Chair of Plastics Technology, Faculty of Mechanical Engineering, TU Dortmund University, Leonhard-Euler-Straße 5, 44227 Dortmund, Germany; 3 Institute of Physical Chemistry, Universität Hamburg, Grindelallee 117, 20146 Hamburg, Germany

**Keywords:** polyethersulfone, poly(ethylene glycol), foam extrusion, polymer blends, open-cell foam

## Abstract

Polyethersulfone (PESU), as both a pristine polymer and a component of a blend, can be used to obtain highly porous foams through batch foaming. However, batch foaming is limited to a small scale and is a slow process. In our study, we used foam extrusion due to its capacity for large-scale continuous production and deployed carbon dioxide (CO_2_) and water as physical foaming agents. PESU is a high-temperature thermoplastic polymer that requires processing temperatures of at least 320 °C. To lower the processing temperature and obtain foams with higher porosity, we produced PESU/poly(ethylene glycol) (PEG) blends using material penetration. In this way, without the use of organic solvents or a compounding extruder, a partially miscible PESU/PEG blend was prepared. The thermal and rheological properties of homopolymers and blends were characterized and the CO_2_ sorption performance of selected blends was evaluated. By using these blends, we were able to significantly reduce the processing temperature required for the extrusion foaming process by approximately 100 °C without changing the duration of processing. This is a significant advancement that makes this process more energy-efficient and sustainable. Additionally, the effects of blend composition, nozzle temperature and foaming agent type were investigated, and we found that higher concentrations of PEG, lower nozzle temperatures, and a combination of CO_2_ and water as the foaming agent delivered high porosity. The optimum blend process settings provided foams with a porosity of approximately 51% and an average foam cell diameter of 5 µm, which is the lowest yet reported for extruded polymer foams according to the literature.

## 1. Introduction

Polyarylsulfones, such as polysulfone (PSU), polyethersulfone (PESU) and polyphenylenesulfone (PPSU), are high-performance thermoplastic polymers that are used for many applications where high strength, high temperature and chemical resistance are required [1,2,3,4]. PESU has thermal, structural and chemical stability due to the combination of sulfonyl groups and aromatic rings in its molecular structure [5]. The high-thermal stability of PESU, a desirable property, enables the use of processing temperatures between 320 °C and 400 °C for extrusion and injection molding [4,6]. This high-temperature processing partially accounts for the high material and production costs. Polyarylsulfone foams have been studied for the last couple of decades for use in various applications. Using the method of batch foaming, highly porous microcellular foams, as well as nanocellular foams, have been produced with PESU, PSU, PPSU and their blends [7,8,9,10,11,12,13,14].

Foam extrusion is a continuous process used to obtain foamed polymer extrudate. It is possible to scale-up the method to an industrial level easily. Unlike batch foaming, which relies on the absorption of the foaming agent into the polymer in the semi-solid state over a longer period to ensure cell nucleation, foam extrusion uses physical mixing at temperatures where the polymer softens or is in the melt state.

The main component of foam extrusion is an extruder with an inlet for the foaming agent. Temperature profiles and screw speed are set on the extruder and a feed rate for the physical foaming agent is specified. The foaming agent mixes with the polymer melt within the extruder at significantly higher pressures than ambient pressure. The foaming agent is dispersed and dissolved due to this high-temperature and high-pressure mixing, reducing the viscosity of the melt [15]. The foaming agent nucleates and expands into pores as the material exits the extruder through a nozzle. This expansion is dependent on various parameters, such as the polymer type, foaming agent, nozzle temperature, pressure, etc. The foamed extrudate takes the shape of the nozzle in two dimensions, as the extrusion process is a continuous process [16].

Huang [17] investigated the foaming of PESU using CO_2_ at various melt temperatures and obtained closed-cell foams with an average cell size of 10 µm at the lowest possible processing temperature of 280 °C. Extrusion below this temperature was not possible due to excessive extruder pressures (high viscosity of the melt). 

In previous studies, researchers used polymer blends that delivered higher porosity and smaller cell sizes than their respective homopolymers [14,18,19,20,21]. As the processing temperatures for PESU lie near or above the degradation temperatures of most polymers, it is essential to lower the processing temperature. Poly(*N*-vinylpyrrolidone) (PVP), a water-soluble polymer, results in improved porosity and pore size in foams of blends with PESU manufactured using batch foaming [14,22]. PVP, however, is susceptible to crosslinking at higher temperatures and is not directly processable in an extruder with PESU [23]. Poly(ethylene glycol) (PEG), another water-soluble polymer [24,25], is also a plasticizer [26,27]; i.e., blending a high-temperature resistant polymer with it would lead to lowered processing temperatures. PEG is used in various applications ranging from medicine to industry [28,29]. Similar to PVP, in the manufacturing of membranes using the non-solvent-induced phase separation process, PEG is used as a pore opener [30,31]. The fabrication of open porous foams is of great importance for the fabrication of polymer membranes [14] and for the creation of sorption foams [32]. These technological applications led us to investigate the possibility of obtaining such structures. Thus, a blend of PESU/PEG was selected in this work, as it could potentially produce highly porous foams using foam extrusion. 

Our aim was to produce PESU/PEG blends with PESU as the matrix component that can be processed at significantly lower processing temperatures than pristine PESU. The formation of blends using organic solvents and compounding is well-known, as well as being a common industrial practice. Organic solvents are classified as auxiliary substances—i.e., substances to be reduced or eliminated wherever possible—and are harmful to human health [33,34,35]. Compounding requires the use of high temperatures to melt all polymer components and consumes energy in similar amounts as a foam extruder. Furthermore, this removes the significance of using a plasticizer as a blend component. Material penetration of organic liquids into polymers has been successfully proven by Gutmann et al. [36] and, as low-molecular-weight PEG is a liquid at room temperature, absorption of PEG into the PESU matrix was pursued. In this way, we prepared blends of PESU with low-molecular-weight PEG without using melt-state compounding or organic solvents.

Out of three low-molecular-weight PEGs, the one in which no crystalline formation was detected and which had the lowest viscosity at near-room temperature was selected and blended with PESU in various proportions. The processibility of these blends in foam extrusion was determined using polymer characterization techniques, such as thermal analysis and rheology. Using foam extrusion, foams were manufactured from the selected blends with various process settings and the effects of blend composition, foaming agent type and nozzle temperature were studied. An annular slit nozzle was selected for extrusion in order to create hollow-fiber-geometry extrudates. This would enable the use of these foams as hollow-fiber membranes in separation applications in the future.

The use of CO_2_ as a foaming agent, especially in the supercritical state, to achieve porous foams in both batch foaming and foam extrusion is widely accepted and studied by researchers [17,37,38,39,40,41,42,43,44,45,46]. Sorption measurements have been conducted to understand the influence of blend composition on CO_2_ diffusion [44,47]. The use of water along with CO_2_ as co-foaming agents increases the porosity of foams and provides smaller cell sizes than the foams obtained when only using CO_2_ as a foaming agent [13,14,48]. Evans et al. observed a twentyfold decrease in the viscosity of polyamide in extrusion when superheated water was used [49]. We used CO_2_ and water together as foaming agents and confirmed their better performance in processing and foam formation in comparison to foams manufactured using only CO_2_. Using CO_2_ and water together as foaming agents delivered more uniform foams than only CO_2_. Evaluation parameters that provide open-celled foams, such as average cell size and porosity, the optimum blend composition and process settings, were identified. 

## 2. Experiment

### 2.1. Materials and Methods

In this study, all polymers used were commercial grade. The type of PESU was selected based on previous foaming studies [10,14,17]. PESU homopolymers, BASF Ultrason^®^ E 3010 (PESU E 3010) in the form of granules and BASF Ultrason^®^ E 3020 P (PESU E 3020 P) in the form of flakes were kindly provided by BASF SE (Ludwigshafen, Germany). As PEG in liquid form was desired, low-molecular-weight PEG 200, 400 and 600 were obtained from Sigma-Aldrich (St. Louis, MO, USA) in liquid form.

**Figure 1:**
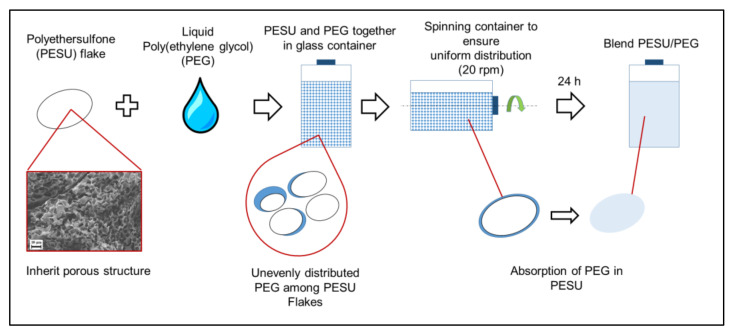
Schematic representation of the method followed for the blending of PEG with PESU.

PESU/PEG blends were produced by adding liquid PEG to PESU flakes in a 5 L cylindrical glass container. The required proportions of PESU and PEG were added in small portions at a time such that the net mass of the mixture added at once was 50 g. Specifically, to prepare the PESU/PEG 80/20 blend, 10 g of PEG was added to 40 g of PESU, summing up to a total of 50 g in the 5 L container. This addition was repeated 32 times, resulting in a total of 1.6 kg of polymer, and the container was filled approximately up to 4 L, leaving 1 L of air in the container after closing. Not filling the container completely allowed free movement of the mixture during rotation. Following that, the glass container was closed and placed on mechanical rollers at 20 rpm for 24 h. This facilitated the uniform distribution of the PEG around the PESU flakes and the thorough mixing of the components. This method can be graphically interpreted from Figure 1. 

The mixture was dried for a minimum of 24 h at 50 °C under vacuum before use. For foam extrusion, the blended flakes were used directly after drying, whereas for material characterization, the blended flakes were ground into powder using a grinder and particles < 350 µm were filtered using an industrial sieve.

The blend formulations, along with the nomenclature, can be found in Table 1. Here, only blends with PEG 200 are shown due to the selection of this molecular weight based on the material characterization, as discussed later.

### 2.2. Material Characterization

Gel permeation chromatography (GPC) was performed in dimethylacetamide using 5 µ PSS SDV gel columns (PSS GmbH, Mainz, Germany) at a flow rate of 1 mL min^−1^ (VWR-Hitachi 2130 pump) and 50 °C. For the detection of the concentration, a Waters UV photometer (typically operated at λ = 254 nm) and a Waters 2410 refractive index (RI) detector (λ = 930 nm) were used. A Waters 717 autosampler with an injection volume of 60 µL was used for the injection of samples. The raw data were analyzed using the PSS WinGPC Unity software package (PSS GmbH, Mainz, Germany). To calculate the apparent average molecular weight and distribution, polystyrene standards (PSS GmbH, Mainz, Germany) calibration was used.

Differential scanning calorimetry (DSC) was undertaken using a DSC 1 calorimeter (Mettler Toledo, Gießen, Germany) and analyzed with the software STARe SW 16.20 (Mettler Toledo, Gießen, Germany). An aluminum pan with a capacity of 40 μL was filled with approximately 10 mg of polymer and closed with a mono-perforated lid. Heating–cooling–heating cycles were implemented at a heating rate of 10 K min^−1^ in a nitrogen atmosphere. The first two temperature intervals for PEG, PESU and PESU/PEG blends were –130 to 100 °C, 25 °C to 260 °C and –130 to 180 °C, respectively. For PESU, the glass transition temperature was determined by evaluating the second heating interval. For PEG and PESU/PEG blends, a third heating–cooling cycle was used from −130 to 260 °C. The heating rate for this cycle was set to 30 K min^−1^ to obtain a pronounced glass transition signal [50]. 

For rheological measurements of PEG, 0.67 mL of the liquid polymer was measured in a cone-plate geometry with an Anton Paar MCR 502 rheometer (Anton Paar, Graz, Austria). A shear amplitude of 5% was applied and viscosities were measured in the angular frequency range from 0.01 to 100 rad/s. 

For rheological measurements of polymers in a glassy state at room temperature, cylindrical samples (8 mm diameter, 2 mm thickness) were prepared using compression molding. A hot press (Paul-Otto Weber, Remshalden, Germany) was used. The temperatures chosen for PESU and PESU/PEG blends were 270 °C and 200 °C, respectively. Samples that were free from defects, such as dents, weldlines, air-bubbles, scratches, etc., resulting from compression molding were used. Rheological measurements were carried out on an Anton Paar MCR 502 rheometer (Anton Paar, Graz, Austria) with a plate–plate geometry. Frequency sweeps in the frequency range between 0.01 and 100 rad/s were carried out at 260, 280, 300 and 320 °C for PESU and 160, 180, 200, 220 and 240 °C for the blends. The frequency sweeps started at the highest frequency. 

Absorption of PEG 200 into PESU was observed by immersing samples of PESU into PEG 200 in an evacuated nitrogen environment at room temperature for 24 h. Then, 1 mm thick compression-molded samples with a diameter of 24 mm were used after drying under a vacuum for 24 h. The mass of the samples was measured before immersion into PEG 200 and after 24 h in PEG 200 using a weighing scale. Before measurement, the samples were dried clean using Kimtech^®^ Science Precision Wipes (Irving, TX, USA) and blow-dried using pressurized nitrogen gas [36].

Sorption experiments were carried out with a IsoSORP^®^ Static gravimetric sorption analyzer from Rubotherm (Bochum, Germany). Flat sheet samples (diameter: 14 mm, thickness: 0.5 mm) were prepared using compression molding. For compression molding, the blend flakes were added to a mold with four voids of the required dimensions and the mold was inserted into a hot press (Paul-Otto Weber, Remshalden, Germany) to be subjected to 200 °C for 10 min. A vacuum was applied from the fourth minute and a force of 60 kN from the sixth minute. The mold was then removed and allowed to cool down towards room temperature when the samples were removed. The thicknesses of the isotropic flat sheet samples were measured with a DELTASCOPE^®^ FMP10 digital micrometer (Fischer, Sindelfingen, Germany). The densities of the flat sheet samples were estimated with the buoyancy method using an Excellence XP105DR analytical balance (Mettler Toledo, Gießen, Germany) and auxiliary liquid FC-770 (3M, Saint Paul, MN, USA), as described elsewhere [51]. The measurement protocol for the sorption experiments and the interpretation of the results were undertaken following previous studies [51,52,53]. All samples were dried in a vacuum for 48 h. A CO_2_ pressure of 50 bar was applied and samples of selected polymers were measured at 35 °C, 50 °C and 75 °C. The weight concentration of CO_2_ per gram of polymer was measured and the data points were smoothed using an adjacent averaging method in Origin (OriginLab, Northampton, MA, USA). The diffusion coefficient ***D_T_*** at temperature ***T*** was calculated by fitting the kinetic sorption curve using the theory of Fickian diffusion following the equation below [54,55]:

(1) $$\frac{M_{t}}{M_{\infty }}=4\sqrt{\frac{D_{T}t}{\pi l²}}$$

where:

$M_{t}$ = mass of gas absorbed by sample at time ***t***;

$M_{\infty }$ = mass of gas absorbed by sample at time ***t*** → ∞; i.e., equilibrium;

l = thickness of the cylindrical sample.

Values used for $\frac{M_{t}}{M_{\infty }}\;$ were lower than 0.6.

The apparent density $\rho_{b}\;$ of the polymer granules and flakes was measured by filling a graduated cylinder (Hirschmann EM Techcolor, Erberstadt, Germany) up to 10 mL with the respective polymers without compression. The mass of the polymer M was measured and the apparent density was calculated.

### 2.3. Foam Extrusion

**Figure 2:**
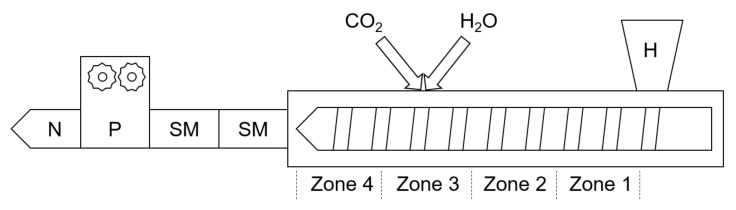
Illustration of the foam extruder setup; H = hopper, SM = static mixer, P = melt pump, N = nozzle.

An Extrusiograph 19/25D single-screw extruder (Brabender GmbH & Co. KG, Duisburg, Germany) was used for foam extrusion. This extruder was coupled with two static mixers with diameters of 2 cm each and a combined length of 16 cm. A melt pump maintained a speed of 10 rpm for all experiments. An annular slit nozzle with a 2 mm outer diameter and 1 mm inner diameter was used with the aim of preparing hollow fiber specimens. The extruder configuration is shown in Figure 2. The temperatures and pressures in the extruder and components were constantly monitored using WINExt software (Brabender Technologies GmbH, Duisburg, Germany). Pressure sensors were present in zone 3, zone 4 and before the nozzle. 

Foaming agents, CO_2_ and water were pressurized using two separate high-pressure syringe pumps (Teledyne ISCO, Thousand Oaks, CA, USA). Water and CO_2_ were injected at zone 3 of the extruder through different inlets on the same longitudinal point of the extruder’s axis, separated by 90° on the extruder barrel. The source of CO_2_ was a dip-tube bottle (99.995% purity, Linde PLC, Dublin, Ireland) and ultrapure water was used. 

Extruder temperatures approximately 100–130 °C higher than the observed glass transition temperatures for the respective blends were set to obtain better mixing due to lower viscosity. Table 2 shows the extruder temperatures for the materials that could be processed in the extruder.

For the trials conducted to study the effect of nozzle temperature on the foaming behavior, the screw speed was set to 10 rpm, and the feed rates of CO_2_ and water were both 0.5 mL/min.

At the beginning of each trial, the nozzle temperatures were equal to the extruder temperatures and CO_2_ and water were injected after ensuring that a constant extrudate was obtained from the nozzle at 10 rpm without any unstable extruder pressures. To observe the effects of the changed extruder setting, the extruder was allowed to run for 20 min before collecting samples. This ensured that the collected sample corresponded to the given settings. To observe the effect of nozzle temperature on the foam, the samples were collected for nozzle temperatures moving from the highest to the lowest possible nozzle temperature. 

### 2.4. Foam Characterization

The morphologies of the polymer, polymer blend and their foams were examined using scanning electron microscopy (SEM) on a Merlin microscope (Carl Zeiss AG, Oberkochen, Germany) at an acceleration voltage of 3 kV. Extrudates were cross-sectioned using liquid nitrogen and sputter-coated with approximately 2 nm of platinum [56]. The average cell size was measured for selected foams using the measurement tool in Photoshop CS6 (Adobe, San Jose, CA, USA) from the scanning electron micrographs. The porosity was measured for selected foams from the SEM micrographs by measuring the number of pixels occupied by visible cells and calculating the percentage versus the total number of pixels in the micrographs. Three micrographs were measured per sample. 

Tensile tests were performed at room temperature using a Zwick Roell Z020 (Zwick Roell, Ulm, Germany) and a load cell of 1 kN. Extrudates with a length of 110 mm were used. The tests were operated and evaluated using the program TestXpert III (Zwick Roell, Ulm, Germany), and data on the true stress and the nominal strain were obtained. Three samples with each process setting were measured and an average curve was calculated until breakpoint. FTIR spectroscopy was performed with the blend and its extruded foams using a Bruker Alpha-P platinum attenuated total reflector equipped with a diamond head (Bruker, Billerica, MA, USA). The measurements were performed by recording 32 scans with a resolution of 4 cm^−1^ within a spectral range of 400–4000 cm^−1^ [57].

The dye uptake test was performed by dipping a 20 mm long extrudate into a 0.1 mg solution of 1-1 methylene in ethanol. The change in the color of the extrudate to blue indicated the uptake of the solution into the extrudate.

## 3. Results and Discussions

**Figure 3:**
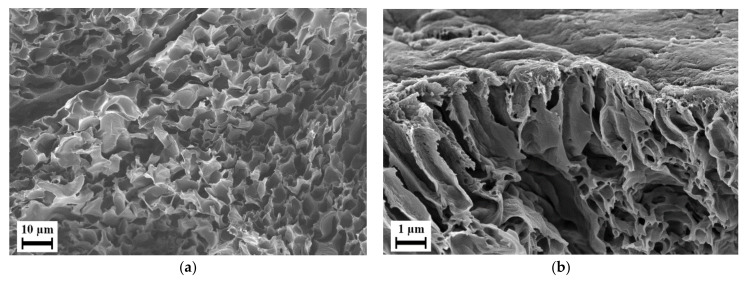
Scanning electron micrograph of PESU E 3020 P flake: (**a**) cross-section, (**b**) surface morphologies.

Although PESU E 3010 was directly usable in the extruder, we selected PESU E 3020 P for blending with PEG due to its porous structure, as seen in Figure 3. 

The average molecular weights and the molecular weight distributions of PESU E 3010 and E 3020 P were examined using GPC and determined to be similar (see the Supplementary Materials). According to the material manufacturer, the third numbers in the material names (1 and 2) indicate granules and flakes, respectively, while the suffix P in PESU E 3020 P denotes the material’s intended use for solution preparation [4]. 

Although low-viscosity polymers can be mixed with a high T_g_ polymer in a single phase using a compounding extruder [58], since we used porous PESU flakes, we could obtain the absorption of the liquid PEG in an easier way. Therefore, low-molecular-weight PEG 200, PEG 400 and PEG 600 were selected as possible candidates for blending with PESU due to their liquid state at room temperature. The capillary action of this porous structure allowed for liquid PEG to enter the flakes and facilitated the absorption of PEG into the PESU matrix. The phenomenon of absorption was confirmed with an absorption test on compression-molded samples, where, for a surface area of 452.38 mm^2^ (sample mass 0.72 g), the mass uptake after one day of immersion in liquid PEG was 0.17%. Due to the porous structure, the active area for material penetration increased enormously, as the pore sizes were in the range of a few micrometers. This significantly increased the ratio of the surface area of contact between PEG and PESU to the volume of PEG. Therefore, PESU/PEG blends were formed. 

**Figure 4:**
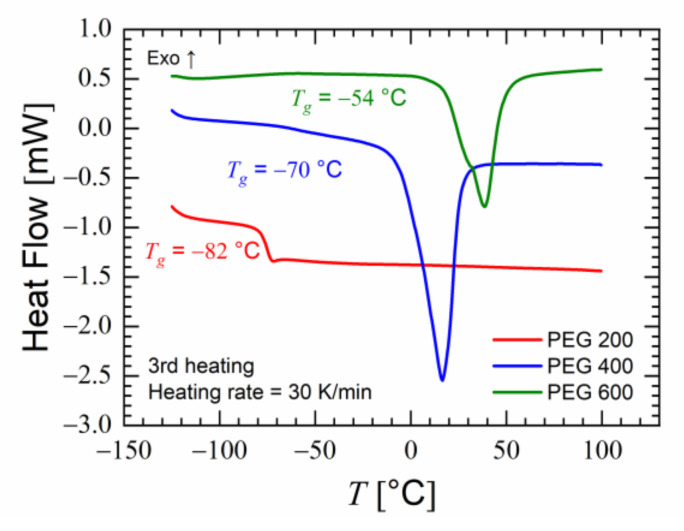
Heat flow measured using differential scanning calorimetry (DSC) during the third heating cycle of PEG 200, 400 and 600.

The viscosities of PEG 200, PEG 400 and PEG 600 at 25 °C were measured as 55, 98, and 190 mPa s, respectively. As we made use of the porous structure of PESU flakes to assist with the absorption of liquid PEG, the lowest viscosity was favored. As seen in Figure 4, PEG 400 and PEG 600 demonstrated melting peaks during the third heating cycle of the DSC. These melting peaks indicated the presence of crystallinity in PEG 400 and PEG 600. As crystallinity is not desired in polymer foaming [59], PEG 400 and PEG 600 were not selected for further investigation. PEG 200 had the lowest viscosity, a low glass transition temperature and did not indicate signs of crystallinity; therefore, it was chosen for blending with PESU. Compression-molded samples of PESU/PEG200 blends were opaque and white in color. This provided our first impression that complete miscibility would not occur for this blend comprising transparent amorphous polymers [60]. 

**Figure 5:**
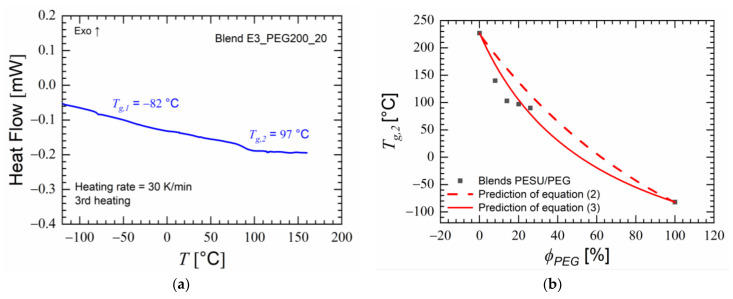
(**a**) Heat flow measured using differential scanning calorimetry (DSC) during the third heating cycle of blend E3_PEG200_20 showed the occurrence of two glass transitions; (**b**) observed second glass transitions (*T_g,2_*) of PESU/PEG blends versus weight percentage of PEG and the predictions from Equations (2) and (3).

Figure 5a shows the third heating cycle of the DSC for the blend E3_PEG200_20. The glass transition of PEG 200 was visible in the blend at −82 °C and a second glass transition was seen at 97 °C. This appearance of a new glass transition temperature that did not belong to any of the polymer components suggested the formation of a miscible polymer blend. This supported the hypothesis that PEG would be absorbed into the PESU matrix after being absorbed on a macro scale by the capillary effect of the porous PESU flakes. Above 160 °C, the heat flow measurement exhibited noise, which hindered the observation of the thermal behavior of the blend around the glass transition temperature of PESU; i.e., 227 °C. This noise occurred during the DSC measurements, likely because the boiling point of PEG 200 lying at 200 °C under atmospheric pressure [61] and the known leaching of PEG from the blend matrix [62]. For blends of fully miscible polymers, the expected glass transition temperature for each mass fraction can be predicted using the Couchman equation (Equation (2)) [63] and the Fox equation (Equation (3)) [64,65]. The glass transition temperatures and the changes in heat capacities from the glassy to the rubbery state in the homopolymers were used in the equations.

(2) $$ln\left(T_{g}/T_{g,I}\right)=\frac{w_{II}\Delta c_{p,II}\ln\left(T_{g,II}/T_{g,I}\right)}{w_{I}\Delta c_{p,I}+w_{II}\Delta c_{p,II}}$$

(3) $$\frac{1}{T_{g}}=\frac{w_{I}}{T_{g,I}}+\frac{w_{II}}{T_{g,II}}$$

where

***w_I_*** = mass fraction of polymer I;

***c_p,I_*** = heat capacity of polymer I;

***T_g,I_*** = glass transition temperature of polymer I;

***w_II_*** = mass fraction of polymer II;

***c_p,II_*** = heat capacity of polymer II;

***T_g,II_*** = glass transition temperature of polymer II. 

Predictions of Equations (2) and (3) did not accurately predict the glass transition temperatures of the blends at various weight compositions. Compliance with these equations would indicate the formation of homogeneous, single-phase polymer blends [14,64,66]. However, the glass transitions observable in Figure 5b were close to the values predicted by Equation (2), and their decrease with an increase in PEG content provided an indication of some degree of miscibility between the two polymers. The evaluation and quantification of miscibility were undertaken by analyzing the rheological measurements of the blends. 

**Figure 6:**
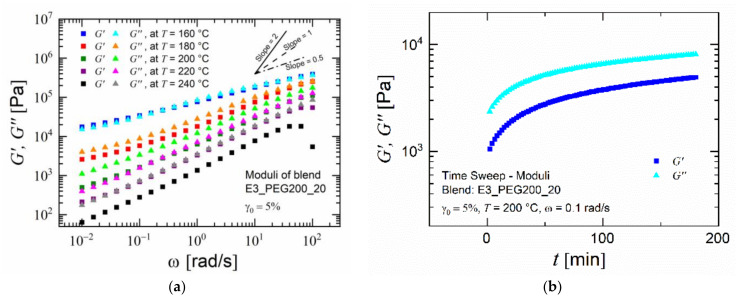
Rheological results for blend E3_PEG200_20: (**a**) frequency sweep: magnitude of moduli versus angular frequency at various temperatures; (**b**) time sweep: magnitude of moduli versus time at 200 °C.

Figure 6a shows that the complex and loss moduli increased in a similar way versus angular frequency for blend E3_PEG200_20. Their slopes lay between 0.35 and 0.75. This behavior cannot be described with the Maxwell model where $G^{\prime }\propto \omega^{2}\;\mathrm{and}\;G^{\prime \prime }\propto \omega$ hold in the terminal regime. These results correspond to power laws, with both the storage and loss moduli following the power laws $G'\propto G''\propto \omega^{0.5}$, indicating microphase separation or another multiphase complex material in the gel state [67]. The data shown in Figure 6a and in Figure S2a–c in the Supplementary Materials for the PESU/PEG blend at a temperature of 160 °C indicate slopes near 0.5 already at high frequencies where evaporation was negligible. This was also an indication that the polymers were not fully miscible [68,69,70,71].

**Figure 7:**
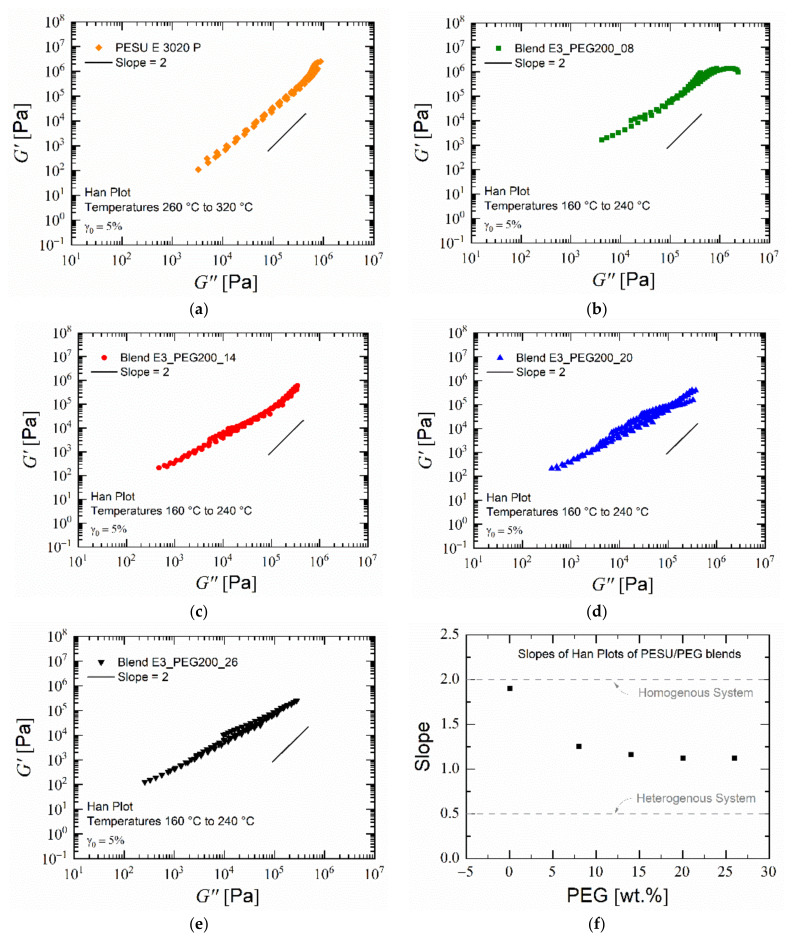
(**a**–**e**) Han plots of PESU and blends PESU/PEG200; (**f**) slopes of Han plots.

As all the rheological measurements were carried out, moving from the highest frequencies to the lowest frequencies, the sample was subjected to its corresponding temperature for approximately 1.5 h. Exposure to temperatures near ± 40 °C of the evaporation temperature of PEG 200 accelerated the leaching of PEG from the samples [72]. This resulted in more accurate values at the highest frequencies, which were relevant for assessing the performance during extrusion, as discussed further below. During the measurements at lower frequencies, the blend samples had a lower net amount of PEG within them than at the previous frequency. This was confirmed by performing time-sweep experiments (Figure 6b). The storage and loss moduli increased equally over time when exposed to a measurement temperature of 200 °C. Plotting a master curve using the time–temperature superposition principle for the blends would not have resulted in accurate values due to the presence of two glass transition temperatures, which indicated heterogeneity, and the large difference between them (>100 K) [73,74]. Rheological measurements at higher temperatures were not pursued due to the rapid evaporation of PEG, which would have resulted in a significant change in the polymer composition over the course of the measurement, thus providing misleading values. The frequency-dependent storage modulus and the loss modulus at various temperatures for PESU and the selected blends were plotted against each other, resulting in a “Han plot” [75]. The resulting plot points were consolidated into a linear fitted curve for lower values of the loss modulus for which the slope values were found. Theoretically, a value of 2 in the low-frequency range indicates a completely homogenous polymeric system, and lower values tend to indicate a non-homogenous mixture [76,77,78,79,80]. As seen in Figure 7, PESU, being a homopolymer, exhibited a slope value near 2. The blend with 8% PEG had a slightly higher slope than the rest of the blend compositions, which had nearly equal slopes around 1.1. Therefore, based on these values and the thermal analysis, it was concluded that PESU and PEG 200 could form a partially miscible system or an even more complex system, such as a gel state. 

**Figure 8:**
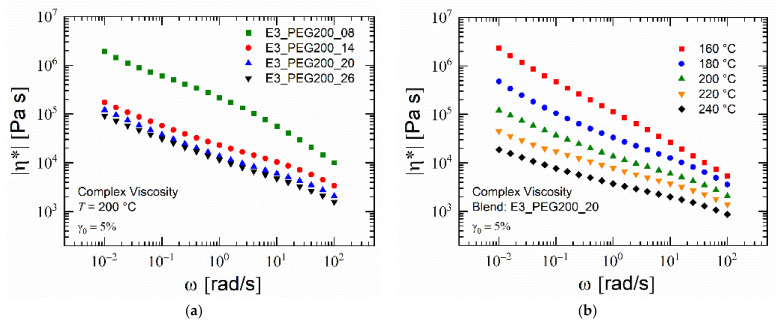
Magnitude of complex viscosity: (**a**) PESU/PEG200 blends at 200 °C; (**b**) blend E3_PEG200_20 at various temperatures.

Rheological analysis carried out on the PESU/PEG blends showed a decrease in complex viscosity with an increase in PEG content at a given temperature, as seen in Figure 8a. The difference between the viscosities of the blend with 8% PEG and the blend with 14 % PEG was the largest, whereas the difference between the blend with 20% and 26% was the smallest. These differences were similar to those seen in glass transition temperatures in DSC measurements. This provided an indication that, above a certain percentage between 20% and 26%, PESU was fully saturated with PEG and excess PEG formed a coexisting phase. This was seen in the results for the frequency sweep, as the complex viscosities tended to increase with lower frequencies and the viscosity–frequency curves did not resemble those of typical homopolymers [81,82,83]. Applying the Cox–Merz rule, as the frequency was replaced by the shear rate, the values of viscosities at shear rates at 100 s^−1^ tended to lie below 10,000 Pa s [84]. As the data points for this frequency were measured at the beginning, the loss of PEG was minimal and the values confirmed the processibility of these blends in an extruder at 200 °C [82,85,86,87,88,89]. Figure 8b shows the influence of temperature on the viscosity of blend E3_PEG200_20. This indicated the temperature range below which this blend could be processed in the foam extruder. 

**Figure 9:**
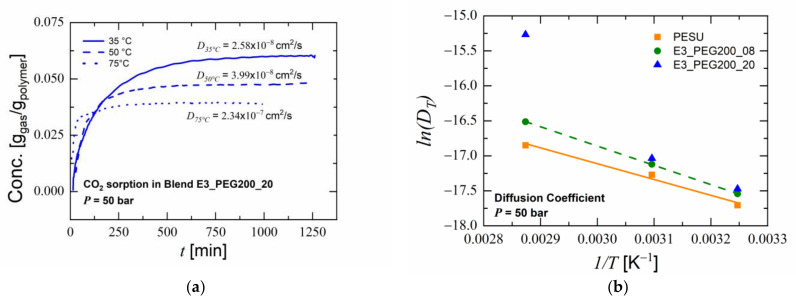
Sorption measurements results: (**a**) concentration of CO_2_ in blend E3_PEG200_20 versus time at various temperatures; (**b**) Arrhenius plot of diffusion coefficients of blends.

The kinetic sorption curves for the glassy polymer below T_g_ can be interpreted as indicating Fickian behavior as a function of the square root of time [90,91]. Similarly, all experiments with pure PESU and with PEG blends resulted in linear plots of the sorption as a function of the square root of time. The influence of PEG content on the CO_2_ sorption properties of PESU/PEG blends was evident at all temperatures, as seen in Figure 9. The diffusion coefficient of the blends increased with the increase in PEG content. As seen in Figure 9a, in blend E3_PEG200_20, the magnitude of the increase in the diffusion coefficient with temperature was much higher than the decrease in the total concentration of CO_2_. By plotting the log of the diffusion coefficient versus the inverse of the temperature, as shown in Figure 9b, the values of PESU and blend E3_PEG200_08 fit linearly and could be defined with the Arrhenius equation [62,92]. Blend E3_PEG200_20, however, yielded too high values for the diffusion coefficient at 75 °C, which did not fit linearly with the other measured temperatures. Plasticization phenomena in the glassy polymer and the existence of rubber phases in the polymer matrix could have caused this high diffusion [55]. This high diffusion coefficient for blend E3_PEG200_20 could be beneficial during foaming to obtain highly porous foams with finer cell sizes [16,36,93].

In the foam extruder, the third zone, called the degassing zone, contained a larger internal volume, as the screw’s inner diameter was the lowest among the zones. Furthermore, CO_2_ and water inlets were present in this zone. For the injection of foaming agents, a high melt pressure should be maintained in this zone. Failure to maintain this pressure causes the foaming agents to find the path of least resistance in the wrong direction of the extruder and escape from the hopper, resulting in blowback. The blended flakes were inserted into the extruder directly. Due to the low apparent density of blend E3_PEG200_08, as seen in Figure S3 in the Supplementary Materials, the difference between the volumes assumed by the blend’s flakes and its melt was higher than for the other blends. Therefore, the same mass of flakes that took up the entire screw volume at the hopper could not fill the complete volume of the degassing zone after melting. Therefore, no melt pressure could be generated at this zone. This caused a reverse flow of foaming agents through the hopper, resulting in blowback. Therefore, foam extrusion could not be carried out with blend E3_PEG200_08. Due to their higher apparent densities, blends E3_PEG200_14 and E3_PEG200_20 were successful in increasing the pressure in the degassing zone and, therefore, foam extrusion was possible. Blend E3_PEG200_26 exhibited separation of PEG from the flakes in the initial zones of the extruder, causing flooding of the extruder with liquid PEG. Some amount of PEG remained within the porous structure of the PESU flakes after mixing and could not be absorbed within the PESU matrix. This suggested that 26% PEG was too high an amount to be absorbed into the PESU porous structure of the flakes. The rheological results shown in Figure 8a indicate that, although the percentage weight difference of PEG in the blends was similar, the difference between the viscosities of the blends with E3_PEG200_20 and E3_PEG200_26 was much lower than the difference between E3_PEG200_14 and E3_PEG200_20. Therefore, absorption of PEG 200 within the PESU matrix was limited to a value slightly above 20% and attained saturation. In this way, the upper and lower limits of the PEG200 concentration in the blend for foam extrusion trials were identified. 

**Figure 10:**
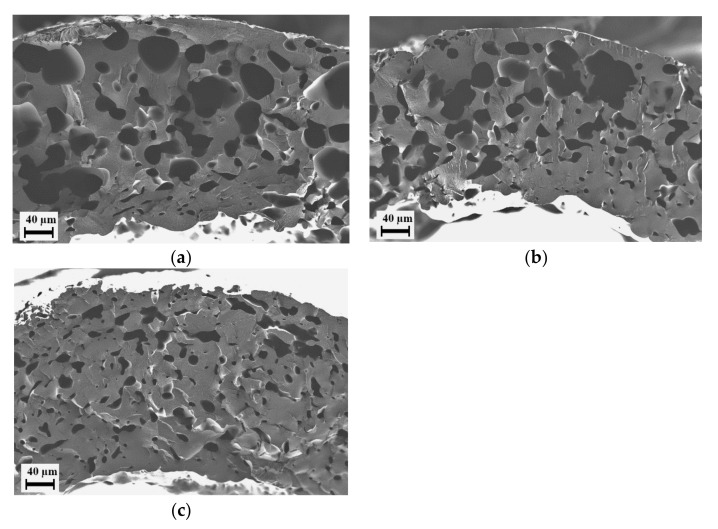
E3_PEG200_20 foams manufactured using only CO_2_ as the foaming agent at various CO_2_ feed rates: (**a**) 0.25 mL/min; (**b**) 0.5 mL/min; (**c**) 0.75 mL/min. Images show close-up views of the cross-section of the extrudate.

In trials of the blend E3_PEG200_20 using only CO_2_ as the foaming agent, the extruder pressures rose higher than 200 bar at a nozzle temperature equal to or lower than 170 °C. Due to limitations in the pressure generation of CO_2_ for injection, it was not possible to conduct trials at these nozzle temperatures. To study the foaming behavior of the blend in the extruder, the nozzle temperature was maintained at 180 °C and CO_2_ feed rates of 0.25, 0.50 and 0.75 mL/min were applied. The effect of the CO_2_ feed rate can be seen in the SEM micrographs in Figure 10. The higher amount of CO_2_ caused more CO_2_ to dissolve into the polymer blend, which resulted in a more swollen polymer phase. However, the pressure at the nozzle was not high enough for this amount of CO_2_ to achieve high nucleation and, subsequently, higher porosity. The foam created using only CO_2_ as the foaming agent was underwhelming compared to the extruded PESU foam [17]. The CO_2_ feed rate of 0.5 mL/min was selected for further experiments since the pore size and porosity were comparatively acceptable, while the operation of the extruder was stable. 

**Figure 11:**
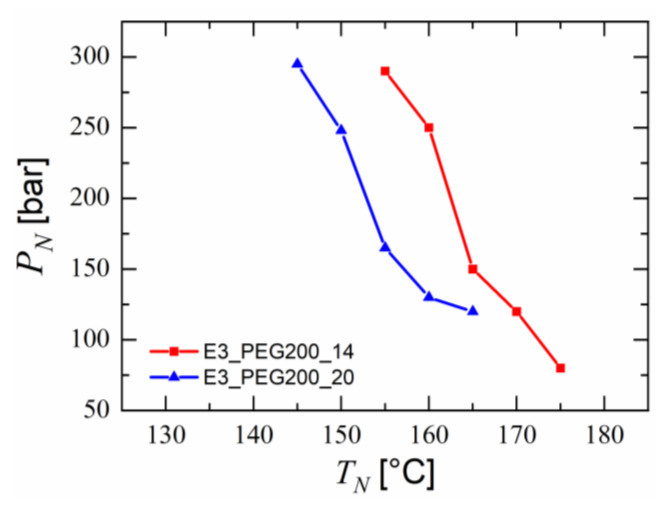
Pressure measured before the nozzle (10 cm) of the foam extruder for PESU/PEG blends at respective nozzle temperatures.

The introduction of water along with CO_2_ decreased the viscosity of the melt in the entire extruder. It was possible to process the blend E3_PEG200_20 with CO_2_ and water as foaming agents at lower nozzle temperatures up to 145 °C. This agreed well with the results from Evans et al. [49], who found a twentyfold decrease in the viscosity of melts due to the introduction of superheated water into the melt system. The effect of nozzle temperature on the foam quality was studied by injecting equal amounts of CO_2_ and water into the foam extruder, as it was identified as one of the most influential extruder settings for the foam quality due to cell nucleation taking place there [16,42,45,94,95,96,97]. This was studied for all processable blends; i.e., blends E3_PEG200_14 and E3_PEG200_20. Lower nozzle temperatures led to an increase in the pressure measured in the extruder near the nozzle, as shown in Figure 11. The difference between the pressures of the two blends correlated qualitatively with the difference between their viscosities, as shown in Figure 8a. The same effect on the pressures was expected if the extruder temperatures were set similarly to the nozzle temperatures. This validated our approach of analyzing the rheological results to predict the performance of the extruder. The measurement of pressure took place approximately 10 cm before the nozzle exit. The pressure in the extruder decreased along the longitudinal axis towards the melt exit in the nozzle and was, therefore, lower than the pressure at the pressure-measuring site near the nozzle [89]. Nevertheless, only the nozzle temperatures that led to a pressure lower than 300 bar were used due to safety precautions.

**Figure 12:**
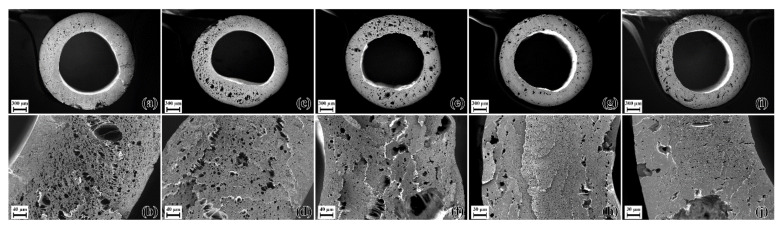
E3_PEG200_14 foams at various nozzle temperatures (T_N_): (**a**,**b**) T_N_ = 155 °C; (**c**,**d**) T_N_ = 160 °C; (**e**,**f**) T_N_ = 165 °C; (**g**,**h**) T_N_ = 170 °C; (**i**,**j**) T_N_ = 175 °C. The images of the entire cross-sections of the extrudates are given, along with close-up views of the cross-sections of the extrudate.

**Figure 13:**
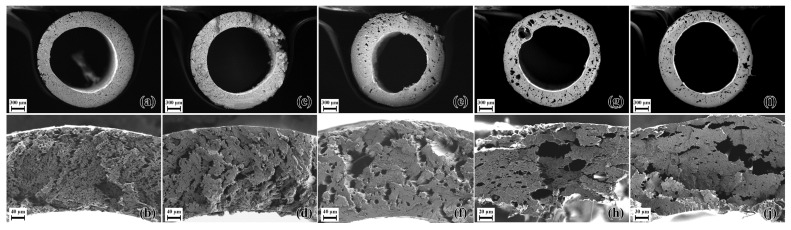
E3_PEG200_20 foams at various nozzle temperatures (T_N_): (**a**,**b**) T_N_ = 145 °C; (**c**,**d**) T_N_ = 150 °C; (**e**,**f**) T_N_ = 155 °C; (**g**,**h**) T_N_ = 160 °C; (**i**,**j**) T_N_ = 165 °C. The images of the entire cross-sections of the extrudates are given, along with close-up views of the cross-sections of the extrudate.

**Figure 14:**
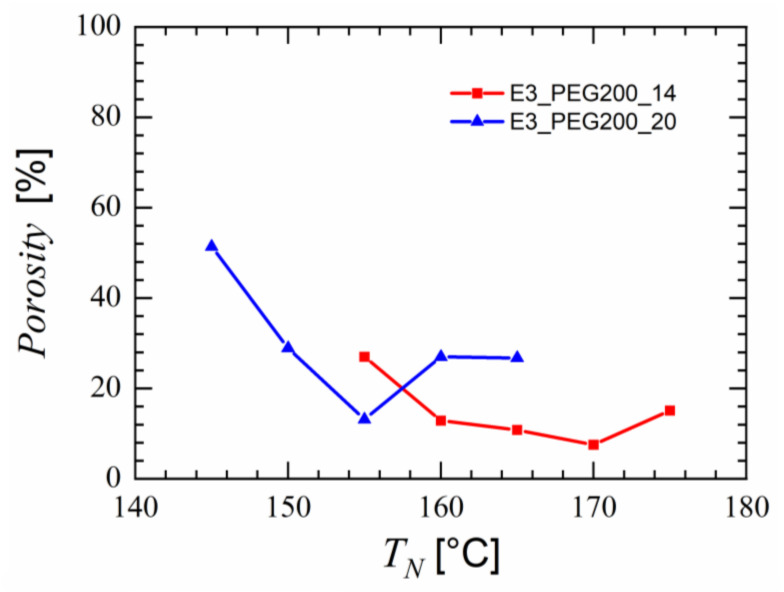
Porosities of foams obtained from the blends E3_PEG200_14 and E3_PEG200_20 at different nozzle temperatures during foam extrusion.

Blend E3_PEG200_14 was processed in the foam extruder at 230 °C and the effect of nozzle temperature was studied between 155 °C and 175 °C, whereas blend E3_PEG200_20 was processed in the foam extruder at 200 °C and the effect of nozzle temperature was studied between 145 °C and 165 °C. Comparing the SEM micrographs in Figure 12 and Figure 13, blend E3_PEG200_20 provided better foam than blend E3_PEG200_14 at lower nozzle temperatures. The effect of nozzle temperature can be seen in both cases, with lower nozzle temperatures yielding higher nucleation and porosity. The lowest nozzle temperature for each blend provided the smallest cell size and highest porosity, as seen in Figure 14. The porosity at higher nozzle temperatures for both materials was slightly higher than the lower adjacent temperature due to the formation of large pores that were essentially macro-cellular in nature. This occurred as the higher nozzle temperature led to lower viscosity, causing the nucleated pores to coalesce and form larger pores in the extrudate. This explains the increases after 155 °C and 170 °C for E3_PEG200_14 and E3_PEG200_20, respectively. At 145 °C, blend E3_PEG200_20 yielded uniform microcellular foam with an average cell size of 5 µm and a porosity of 51%. This average cell size was smaller than those found in the literature on extruded foams obtained from pristine polymers, as the lowest average cell size for the polystyrene foams produced by Han et al. was approximately 7 µm [41,98]. Lee et al. achieved an average cell size of 5 µm using foam extrusion, but they used LDPE/clay nanocomposites [99]. The porosity value was, however, lower compared to the values in the literature [43,100]. The cells seen in Figure 13b appear to be interconnected, and the foam can be classified as open-celled foam [37]. Furthermore, the dye uptake test revealed uptake of the solution of 1-1 methylene blue in ethanol due to the color changing from white to blue. This indicated that the capillary effect enabled the absorption of the solution into the foam, thus confirming the open cellularity [101,102,103].

**Figure 15:**
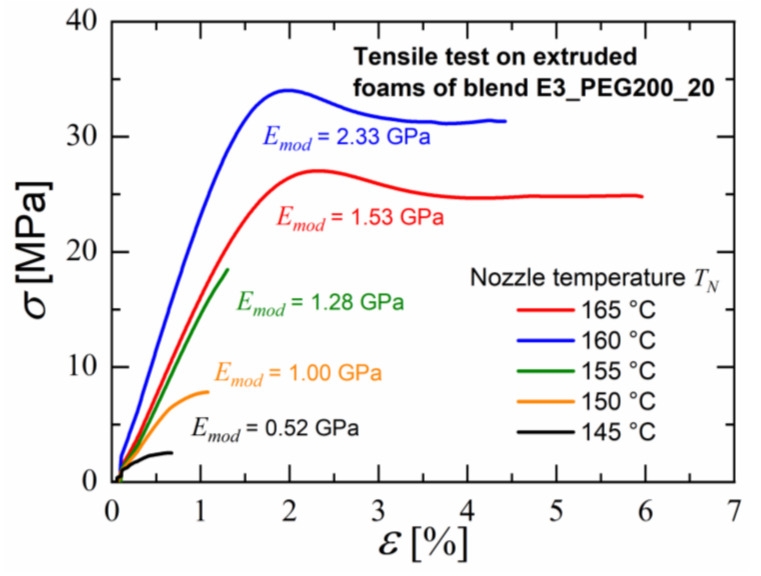
Tensile tests of foamed extrudates of blend E3_PEG200_20 at different nozzle temperatures during foam extrusion.

By carrying out tensile tests on the foamed extrudates of blend E3_PEG200_20, the effect of nozzle temperature on the mechanical properties of the foams was observed. Stress versus strain curves, as seen in Figure 15, indicated that a decrease in nozzle temperature led to a lower E modulus while decreasing the tensile strength. However, an exception occurred for the nozzle temperature 160 °C, which led to a higher tensile strength than 165 °C. This can be correlated with the increase in the overall porosity due to larger pores, as seen in Figure 14, but, at the same time, more volume was achieved without pores. This mechanical analysis also showed that an increase in porosity increased the ductility but also decreased the tensile strength significantly. 

**Figure 16:**
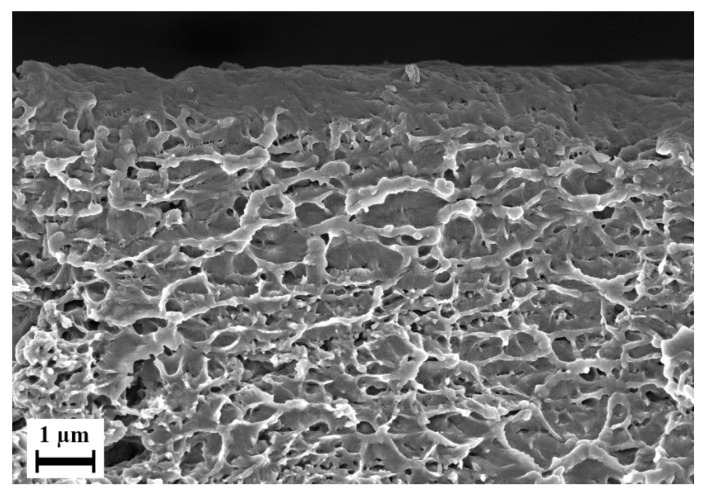
Porous structure observed inside the microcellular foam of the blend E3_PEG200_20.

The cell walls of this foam were porous on their own at the nanocellular level, as seen in Figure 16. This phenomenon has been previously observed in batch foaming in several studies [11,14,104]. The expansion of microcellular cells in foam during foam expansion expresses the tension within the polymer and causes stretching. This stretching leads to fibril-like structures that remain connected to each other and pores are created between them. In a previous work, it was possible to control the pore size and expansion of these structures in PESU/PVP foams manufactured using batch foaming [14]. This was beyond the scope of this work. We suspected that a similar process took place where the bubble growth occurred as the extrudate exited the foam extruder, resulting in stretching of fibrils within the polymer melt and, thus, causing this structure to form. This is the first instance that such a nanocellular structure has been observed in a foam created via extrusion. The pores on the cell walls of microcells were not considered when measuring the average cell size of the complete foams. 

Comparing Figure 10 with Figure 13, the improvement in foam quality due to the usage of water along with CO_2_ as a foaming agent was very significant. This supports the findings of other research, where the usage of water along with CO_2_ led to increased porosity and low pore size [13,14,48]. When water was injected into the extruder, it was in a superheated phase, as the extruder possessed pressures in the range of 100–300 bar and temperatures around 200 °C. Superheated water has a polarity similar to that of organic solvents [105] and, thus, aids in pore formation [49]. The presence of the nanocellular pores, as seen in Figure 15, may also have been observed as a result of the removal of PEG from the polymer matrix due to superheated water, similarly to the phase inversion process, or due to evaporation. The thermal and chemical behavior of the produced extrudate was further investigated.

**Figure 17:**
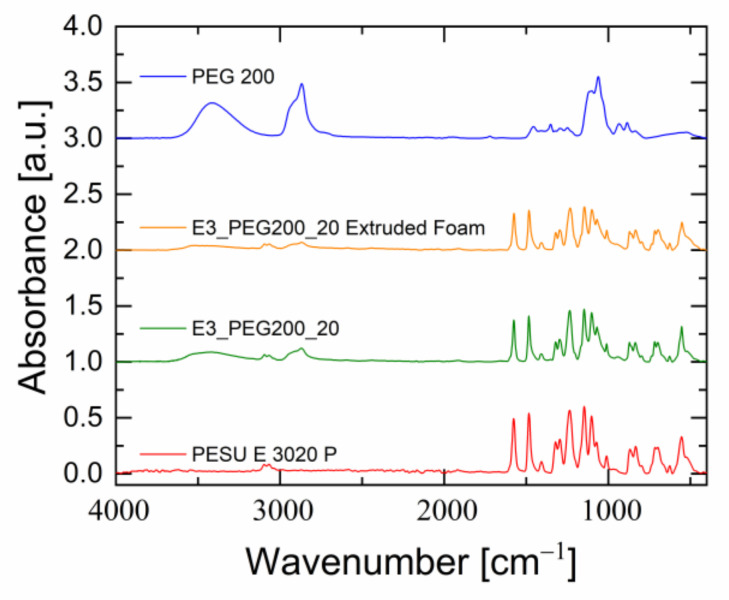
FTIR spectra of PESU E 3020 P, blend E3_PEG200_20, the extruded foam of blend E3_PEG200_20 and PEG200.

The spectroscopical analysis provided some more information regarding the blending of PEG with PESU after the extrusion process. A comparison of the peaks of PESU in the FTIR spectra of blend E3_PEG200_20 and its extruded foam, as shown in Figure 17, indicated no changes in the characteristic vibrations of PESU and PEG. This indicated that no chemical reactions took place. Since the principle peaks of PESU remained similar in both the blend and the extrudate, the chemical stability and resistance of PESU were retained in the extruded foam. Comparing the DSC measurement of the blend’s foam with the blend, an increase of 26 °C was seen after foam extrusion (see Figure S4 in the Supplementary Materials). The amount of PEG appeared to have been reduced, most likely due to evaporation, as the extruder temperatures were near the boiling point of the PEG. This change was also visible in the FTIR spectrum, as the intensities of PEG peaks in the blend’s extruded foam were slightly lower than in the original blend. This also agreed with the observations from the rheological measurements discussed above, since the low molecular weight of PEG enables its leaching from the polymer matrix [72]. This effect would be beneficial for the production of extruded porous hollow fibers based on PESU via foam extrusion.

## 4. Conclusions

PESU/PEG blends were manufactured using the absorption of low-molecular-weight liquid PEG into PESU by taking advantage of the porous structure of the PESU flakes. The polymers were partially miscible, as shown by thermal and rheological measurements. Blends with suitable compositions could be extruded. The sorption of CO_2_ in selected blends was investigated, and it was found that PEG increased the diffusion coefficient of the blend, and it essentially increased with increases in temperature, providing insight into the foaming characteristics of the blends. Blend PESU/PEG 80/20 was processable in a foam extruder at 200 °C; i.e., 150 °C lower than the extruder temperature required for pure PESU. The use of water along with CO_2_ as the foaming agent was confirmed to lower the viscosity of the polymer–foaming agent mixture in the extruder even more than when using only CO_2_ and resulted in highly porous foams with smaller cell sizes. At certain process settings, the PESU/PEG 80/20 blend provided the lowest average pore size of 5 µm and a porosity of 51%. The mechanical properties of certain foams were also evaluated, and it was found that an increase in porosity led to a slight increase in the ductility but, at the same time, a significant loss in tensile strength. FTIR measurements confirmed the retention of the PESU chemical structure in the produced foams, but a certain amount of PEG was lost due to evaporation during the foam extrusion; however, this was not problematic due to the aim of obtaining porous hollow fibers later on. 

## Figures and Tables

**Table 1:** Nomenclature for PESU/PEG blends.

Blend Name	PESU E 3020 P	PEG 200
	Content (wt%)	Content (wt%)
E3_PEG200_08	92	8
E3_PEG200_14	86	14
E3_PEG200_20	80	20
E3_PEG200_26	74	26

**Table 2:** Temperatures set on extruders for selected materials.

Material	*T_ext_*
	(°C)
E3_PEG_14	230
E3_PEG_20	200

## Data Availability

The characterization data are available upon request from the authors.

## Supplementary Materials

The following supporting information can be downloaded at: https://www.mdpi.com/article/10.3390/polym15010118/s1. Figure S1: Gel permeation chromatography for PESU E 3010 and E 3020 P. The molecular weight estimation was based on calibration with polystyrene standards; Figure S2: Rheological investigations of the PESU/PEG200 blends: storage modulus G’ and loss modulus G’’ versus angular frequency at various temperatures for blend (a) E3_PEG200_08, (b) E3_PEG200_14 and (c) E3_PEG200_26; Figure S3: Apparent densities of PESU E 3010 granules, PESU E 3020 P flakes and PESU/PEO blend flakes; Figure S4: DSC third heating cycle for foam of blend E3_PEG200_20; extrusion foamed using CO_2_ and water.

## Nomenclature

**Symbol**	**Parameter**	**Unit**
***T_g_***	Glass transition temperature	K
***w***	Mass fraction of polymer	%
***c_p,_***	Heat capacity of polymer	mW
t	Time	s
$M_{t}$	Mass of gas absorbed by sample at time *t*	g
***M*_∞_**	Mass of gas absorbed by sample at time *t* → ∞; i.e., equilibrium	g
***D_T_***	Diffusion coefficient at temperature ***T***	
$\rho_{apparent}$	Apparent density	g/mL
***T_ext_***	Extruder temperature	°C
***T_N_***	Nozzle temperature	°C
***P_N_***	Nozzle pressure	bar
***G′***	Storage modulus	Pa
***G′′***	Loss modulus	Pa
***ω***	Angular frequency	rad/s
***|*η*|**	Complex viscosity	Pa s

## References

[1] Giesa R., Schmidt H.W., Buschow K.H.J., Cahn R.W., Flemings M.C., Ilschner B., Kramer E.J., Mahajan S., Veyssière P. (2001). High-temperature Stable Polymers. Encyclopedia of Materials: Science and Technology.

[2] Lutz H., Drioli E., Giorno L. (2010). 2.06—Ultrafiltration: Fundamentals and Engineering. Comprehensive Membrane Science and Engineering.

[3] Biernat U. (2018). Threepart Sandwich, One Thermoplastic, Many Possibilities.

[4] BASF (2020). Ultrason® (Psu, Pesu, Ppsu)—The Specialty Plastic for High-Quality Parts.

[5] Tsehaye M.T., Velizarov S., Van der Bruggen B. (2018). Stability of polyethersulfone membranes to oxidative agents: A review. Polym. Degrad. Stabil..

[6] Qi K., Huang R., Cheremisinoff N.P. (1998). 3—Polyethersulfone (PES) and Its Processing. Advanced Polymer Processing Operations.

[7] Krause B., Boerrigter M.E., van der Vegt N.F.A., Strathmann H., Wessling M. (2001). Novel open-cellular polysulfone morphologies produced with trace concentrations of solvents as pore opener. J. Membr. Sci..

[8] Krause B., van der Vegt N.F.A., Wessling M. (2002). New ways to produce porous polymeric membranes by carbon dioxide foaming. Desalination.

[9] Krause B., Diekmann K., van der Vegt N.F.A., Wessling M. (2002). Open nanoporous morphologies from polymeric blends by carbon dioxide foaming. Macromolecules.

[10] Sorrentino L., Aurilia M., Iannace S. (2011). Polymeric Foams from High-Performance Thermoplastics. Adv. Polym. Technol..

[11] Guo H.M., Nicolae A., Kumar V. (2016). Fabrication of high temperature polyphenylsulfone nanofoams using high pressure liquid carbon dioxide. Cell Polym..

[12] Guo H.M., Nicolae A., Kumar V. (2015). Solid-state microcellular and nanocellular polysulfone foams. J. Polym. Sci. Pol. Phys..

[13] Owusu-Nkwantabisah S., Staudt C., Lesser A.J. (2018). Synergy of supercritical CO_2_ and superheated H_2_O for enhanced processability of polyethersulfone towards open cell foams. Polym. Eng. Sci..

[14] Raje A., Buhr K., Koll J., Lilleparg J., Abetz V., Handge U.A. (2022). Open-Celled Foams of Polyethersulfone/Poly(N-vinylpyrrolidone) Blends for Ultrafiltration Applications. Polymers.

[15] Jin F.-L., Zhao M., Park M., Park S.-J. (2019). Recent Trends of Foaming in Polymer Processing: A Review. Polymers.

[16] Lee S.T., Lee S.T., Park C.B., Polymeric Foams (2014). Foam Extrusion.

[17] Huang Q. (2000). Lösemittelfreie Herstellung von Porösen Polymeren Membranen Durch Schaumextrusion. Ph.D. Thesis.

[18] Ruckdäschel H., Gutmann P., Altstädt V., Schmalz H., Müller A.H.E., Müller A.H.E., Schmidt H.-W. (2010). Foaming of Microstructured and Nanostructured Polymer Blends. Complex Macromolecular Systems I.

[19] Bärwinkel S., Bahrami R., Löbling T.I., Schmalz H., Müller A.H.E., Altstädt V. (2016). Polymer Foams Made of Immiscible Polymer Blends Compatibilized by Janus Particles—Effect of Compatibilization on Foam Morphology. Adv. Eng. Mater..

[20] Kong W.-l., Bao J.-B., Wang J., Hu G.-H., Xu Y., Zhao L. (2016). Preparation of open-cell polymer foams by CO_2_ assisted foaming of polymer blends. Polymer.

[21] Haurat M., Dumon M. (2020). Amorphous Polymers’ Foaming and Blends with Organic Foaming-Aid Structured Additives in Supercritical CO_2_, a Way to Fabricate Porous Polymers from Macro to Nano Porosities in Batch or Continuous Processes. Molecules.

[22] Krause B., Wessling M., Göhl H., Storr M., European Patent Office (2020). Membrane and Use Thereof.

[23] Yoshida M., Prasad P.N. (1996). Fabrication of channel waveguides from sol-gel-processed polyvinylpyrrolidone/SiO_2_ composite materials. Appl. Opt..

[24] Raviv U., Klein J., Matyjaszewski K., Möller M. (2012). 2.24—Adhesion, Friction, and Lubrication between Polymer-Bearing Surfaces. Polymer Science: A Comprehensive Reference.

[25] Ibrahim M.S., El-Wassefy N.A., Farahat D.S., Tayebi L., Moharamzadeh K. (2017). 8—Biocompatibility of dental biomaterials. Biomaterials for Oral and Dental Tissue Engineering.

[26] Porter S., Sackett G., Liu L., Qiu Y., Chen Y., Zhang G.G.Z., Liu L., Porter W.R. (2009). Chapter 33—Development, Optimization, and Scale-up of Process Parameters: Pan Coating. Developing Solid Oral Dosage Forms.

[27] Shah H., Jain A., Laghate G., Prabhudesai D., Adejare A. (2021). Chapter 32—Pharmaceutical excipients. Remington.

[28] Hutanu D., Frishberg M.D., Guo L., Darie C.C. (2014). Recent Applications of Polyethylene Glycols (PEGs) and PEG Derivatives. Mod. Chem. Appl..

[29] Dimitrov I., Tsvetanov C.B., Matyjaszewski K., Möller M. (2012). 4.27—Oligomeric Poly(ethylene oxide)s. Functionalized Poly(ethylene glycol)s. PEGylation. Polymer Science: A Comprehensive Reference.

[30] Gronwald O., Frost I., Ulbricht M., Shalmani A.K., Panglisch S., Grunig L., Handge U.A., Abetz V., Heijnen M., Weber M. (2020). Hydrophilic poly(phenylene sulfone) membranes for ultrafiltration. Sep. Purif. Technol..

[31] Hao Y., Liang C., Moriya A., Matsuyama H., Maruyama T. (2012). Visualization of Protein Fouling inside a Hollow Fiber Ultrafiltration Membrane by Fluorescent Microscopy. Ind. Eng. Chem. Res..

[32] Barry E., Mane A.U., Libera J.A., Elam J.W., Darling S.B. (2017). Advanced oil sorbents using sequential infiltration synthesis. J. Mater. Chem. A.

[33] Gronwald O., Weber M. (2020). AGNIQUE AMD 3L as green solvent for polyethersulfone ultrafiltration membrane preparation. J. Appl. Polym. Sci..

[34] Anastas P.T., Warner J.C. (1998). Green chemistry. Frontiers.

[35] Redlich C.A., Beckett W.S., Sparer J., Barwick K.W., Riely C.A., Miller H., Sigal S.L., Shalat S.L., Cullen M.R. (1988). Liver-disease associated with occupational exposure to the solvent dimethylformamide. Ann. Intern. Med..

[36] Gutmann P., Hildebrandt K., Altstädt V., Müller A.H.E. (2010). Foaming of an Immiscible Blend System Using Organic Liquids as Blowing Agents. J. Cell. Plast..

[37] Okolieocha C., Raps D., Subramaniam K., Altstädt V. (2015). Microcellular to nanocellular polymer foams: Progress (2004–2015) and future directions—A review. Eur. Polym. J..

[38] Hwang Y.D., Cha S.W. (2002). The relationship between gas absorption and the glass transition temperature in a batch microcellular foaming process. Polym. Test..

[39] Sauceau M., Fages J., Common A., Nikitine C., Rodier E. (2011). New challenges in polymer foaming: A review of extrusion processes assisted by supercritical carbon dioxide. Prog. Polym. Sci..

[40] Jacobs L.J.M. (2008). Carbon Dioxide as a Sustainable Means to Control Polymer Foam Morphology. Ph.D. Thesis.

[41] Han X., Koelling K.W., Tomasko D.L., Lee L.J. (2002). Continuous microcellular polystyrene foam extrusion with supercritical CO_2_. Polym. Eng. Sci..

[42] Park C.B., Behravesh A.H., Venter R.D. (1998). Low density microcellular foam processing in extrusion using CO_2_. Polym. Eng. Sci..

[43] Chauvet M., Sauceau M., Fages J. (2017). Extrusion assisted by supercritical CO_2_: A review on its application to biopolymers. J. Supercrit. Fluids.

[44] Michaeli W., Heinz R. (2000). Foam extrusion of thermoplastic polyurethanes (TPU) using CO_2_ as a blowing agent. Macromol. Mater. Eng..

[45] Yeh S.-K., Yang S.-H., Han L., Liu H.-Y., Liao Y.-S., Chang Y.-C. (2019). Foam extrusion of polypropylene–rice husk composites using CO_2_ as the blowing agent. J. Cell. Plast..

[46] Mi H.-Y., Jing X., Liu Y., Li L., Li H., Peng X.-F., Zhou H. (2019). Highly Durable Superhydrophobic Polymer Foams Fabricated by Extrusion and Supercritical CO_2_ Foaming for Selective Oil Absorption. ACS Appl. Mater. Interfaces.

[47] Sun H.L., Sur G.S., Mark J.E. (2002). Microcellular foams from polyethersulfone and polyphenylsulfone—Preparation and mechanical properties. Eur. Polym. J..

[48] Schulze M., Handge U.A., Abetz V. (2017). Preparation and characterisation of open-celled foams using polystyrene-*b*-poly(4-vinylpyridine) and poly(4-methylstyrene)-*b*-poly(4-vinylpyridine) diblock copolymers. Polymer.

[49] Evans G.C., Lesser A.J. (2018). Processing polyamides with superheated water. J. Polym. Sci. Part B Polym. Phys..

[50] Papageorgiou G.Z., Bikiaris D.N. (2005). Crystallization and melting behavior of three biodegradable poly(alkylene succinates). A comparative study. Polymer.

[51] Halder K., Khan M.M., Grünauer J., Shishatskiy S., Abetz C., Filiz V., Abetz V. (2017). Blend membranes of ionic liquid and polymers of intrinsic microporosity with improved gas separation characteristics. J. Membr. Sci..

[52] Schulze M., Handge U.A., Rangou S., Lillepärg J., Abetz V. (2015). Thermal properties, rheology and foams of polystyrene-block-poly(4-vinylpyridine) diblock copolymers. Polymer.

[53] Wang J.S., Kamiya Y. (1995). Concurrent Measurements of Sorption and Dilation Isotherms and Diffusivity for Polysulfone Membrane Carbon-Dioxide System. J. Membr. Sci..

[54] Barrer R.M. (1984). Diffusivities in Glassy-Polymers for the Dual Mode Sorption Model. J. Membr. Sci..

[55] Frisch H.L. (1980). Sorption and Transport in Glassy-Polymers—Review. Polym. Eng. Sci..

[56] Höhme C., Filiz V., Abetz C., Georgopanos P., Scharnagl N., Abetz V. (2018). Postfunctionalization of Nanoporous Block Copolymer Membranes via Click Reaction on Polydopamine for Liquid Phase Separation. ACS Appl. Nano Mater..

[57] Georgopanos P., Eichner E., Filiz V., Handge U.A., Schneider G.A., Heinrich S., Abetz V. (2017). Improvement of mechanical properties by a polydopamine interface in highly filled hierarchical composites of titanium dioxide particles and poly(vinyl butyral). Compos. Sci. Technol..

[58] Cassagnau P., Courmont M., Melis F., Puaux J.P. (2005). Study of mixing of liquid/polymer in twin screw extruder by residence time distribution. Polym. Eng. Sci..

[59] Naeini A.T. (2012). Visualization of the Crystallization in Foam Extrusion Process. Theory Comput. Syst. Math. Syst. Theory.

[60] Maruhashi Y., Iida S. (2001). Transparency of polymer blends. Polym. Eng. Sci..

[61] (2021). Safety Data Sheet—Polyethylene Glycol 200 for Synthesis.

[62] Lillepärg J., Georgopanos P., Shishatskiy S. (2014). Stability of blended polymeric materials for CO_2_ separation. J. Membr. Sci..

[63] Couchman P.R. (1987). The Glass-Transition of Compatible Blends. Polym. Eng. Sci..

[64] Fox T.G. (1956). Influence of diluent and of copolymer composition on the glass temperature of a polymer system. Bull. Am. Phys. Soc..

[65] Georgopanos P., Handge U.A., Abetz C., Abetz V. (2016). Influence of block sequence and molecular weight on morphological, rheological and dielectric properties of weakly and strongly segregated styrene-isoprene triblock copolymers. Polymer.

[66] Couchman P.R. (1978). Compositional variation of glass-transition temperatures. 2. Application of thermodynamic theory to compatible polymer blends. Macromolecules.

[67] Sailer C., Weber M., Steininger H., Handge U.A. (2009). Grafting of polyamide 6 on a styrene-acrylonitrile maleic anhydride terpolymer: Melt rheology at the critical gel state. Rheol. Acta.

[68] Asthana H., Jayaraman K. (1999). Rheology of Reactively Compatibilized Polymer Blends with Varying Extent of Interfacial Reaction. Macromolecules.

[69] Stadler R., Freitas L.D., Krieger V., Klotz S. (1988). Influence of the Phase-Separation on the Linear Viscoelastic Properties of a Polystyrene Polyvinyl Methyl-Ether) Blend. Polymer.

[70] Bates F.S. (1984). Block Copolymers near the Microphase Separation Transition. 2. Linear Dynamic Mechanical-Properties. Macromolecules.

[71] Mani S., Malone M.F., Winter H.H. (1992). Influence of Phase-Separation on the Linear Viscoelastic Behavior of a Miscible Polymer Blend. J. Rheol..

[72] Lillepärg J., Georgopanos P., Emmler T., Shishatskiy S. (2016). Effect of the reactive amino and glycidyl ether terminated polyethylene oxide additives on the gas transport properties of Pebax® bulk and thin film composite membranes. RSC Adv..

[73] Urakawa O., Ujii T., Adachi K. (2006). Dynamic heterogeneity in a miscible poly(vinyl acetate)/poly(ethylene oxide) blend. J. Non-Cryst. Solids.

[74] Kalogeras I.M. (2016). Glass-Transition Phenomena in Polymer Blends. Encyclopedia of Polymer Blends.

[75] Han C.D., Kim J. (1987). Rheological Technique for Determining the Order—Disorder Transition of Block Copolymers. J. Polym. Sci. Pol. Phys..

[76] Yu Z.X., Wang J., Li P.H., Ding D.C., Zheng X., Hu C.Q., Gao Z.N., Hu T., Gong X.H., Wu C.G. (2020). Melt Blending Modification of Commercial Polystyrene with Its Half Critical Molecular Weight, High Ion Content Ionomer, Poly(styrene-ran-cinnamic Acid) Zn Salt, toward Heat Resistance Improvement. Polymers.

[77] Walha F., Lamnawar K., Maazouz A., Jaziri M. (2016). Rheological, Morphological and Mechanical Studies of Sustainably Sourced Polymer Blends Based on Poly(Lactic Acid) and Polyamide 11. Polymers.

[78] Luo H.Y., Han H., Chi H.F., Li J.Y., Zhao S.M., Tao Y., Hu H.Q. (2021). Research on the viscous flow transition of styrene-isoprene-styrene triblock copolymer by Rheology. J. Polym. Res..

[79] Tian J., Yu W., Zhou C. (2006). The preparation and rheology characterization of long chain branching polypropylene. Polymer.

[80] Wu D., Huang A., Fan J., Xu R., Liu P., Li G., Yang S. (2021). Effect of blending procedures and reactive compatibilizers on the properties of biodegradable poly(butylene adipate-co-terephthalate)/poly(lactic acid) blends. J. Polym. Eng..

[81] Dunstan D.E. (2019). The viscosity-radius relationship for concentrated polymer solutions. Sci. Rep..

[82] John Vlachopoulos N.D.P., Kontopoulou M. (2012). Basic Concepts in Polymer Melt Rheology and Their Importance in Processing. Applied Polymer Rheology: Polymeric Fluids with Industrial Applications.

[83] Hernández-Alamilla M., Valadez-Gonzalez A. (2016). The effect of two commercial melt strength enhancer additives on the thermal, rheological and morphological properties of polylactide. J. Polym. Eng..

[84] Roland C.M., Mark J.E., Erman B., Roland C.M. (2013). Chapter 6—Rheological Behavior and Processing of Unvulcanized Rubber. The Science and Technology of Rubber.

[85] Vlachopoulos J., Strutt D. The Role of Rheology in Polymer Extrusion. Proceedings of the Extrusion Minitec and Conference: From Basics to Recent Developments.

[86] John Vlachopoulos N.D.P. (2019). Understanding Rheology and Technology of Polymer Extrusion.

[87] Mitsoulis E., Hatzikiriakos S.G. (2021). Rheological Properties Related to Extrusion of Polyolefins. Polymers.

[88] Abeykoon C., Kelly A., Wilkinson A. (2019). Investigation of Thermal Stability of Non-Newtonian Melt Flows.

[89] Han C.D., Villamizar C.A. (1978). Studies on structural foam processing I. The rheology of foam extrusion. Polym. Eng. Sci..

[90] Di Maio E., Iannace S., Mensitieri G., Di Maio E., Iannace S., Mensitieri G. (2021). Chapter 6—Mass transport of low molecular weight compounds in polymers. Supercritical Fluid Science and Technology.

[91] Berens A.R. (1990). Transport of Plasticizing Penetrants in Glassy Polymers. Barrier Polymers and Structures.

[92] Barrer R.M., Rideal E.K. (1939). Permeation, diffusion and solution of gases in organic polymers. Trans. Faraday Soc..

[93] Gendron R. (2004). Thermoplastic Foam Processing: Principles and Development.

[94] Standau T., Castellón S.M., Delavoie A., Bonten C., Altstädt V. (2019). Effects of chemical modifications on the rheological and the expansion behavior of polylactide (PLA) in foam extrusion. e-Polymers.

[95] Shabani A., Fathi A., Erlwein S., Altstädt V. (2021). Thermoplastic polyurethane foams: From autoclave batch foaming to bead foam extrusion. J. Cell. Plast..

[96] Kalia K., Francoeur B., Amirkhizi A., Ameli A. (2022). In Situ Foam 3D Printing of Microcellular Structures Using Material Extrusion Additive Manufacturing. ACS Appl. Mater. Interfaces.

[97] Doyle L. (2022). Extrusion foaming behavior of polybutene-1. Toward single-material multifunctional sandwich structures. J. Appl. Polym. Sci..

[98] Azdast T., Hasanzadeh R. (2021). Increasing cell density/decreasing cell size to produce microcellular and nanocellular thermoplastic foams: A review. J. Cell. Plast..

[99] Lee Y.H., Wang K.H., Park C.B., Sain M. (2007). Effects of clay dispersion on the foam morphology of LDPE/clay nanocomposites. J. Appl. Polym. Sci..

[100] Nikitine C., Rodier E., Sauceau M., Letourneau J.-J., Fages J. (2010). Controlling the structure of a porous polymer by coupling supercritical CO_2_ and single screw extrusion process. J. Appl. Polym. Sci..

[101] Minju N., Jobin G., Savithri S., Ananthakumar S. (2019). Double-Silicate Derived Hybrid Foams for High-Capacity Adsorption of Textile Dye Effluent: Statistical Optimization and Adsorption Studies. Langmuir.

[102] Galzerano B., Cabello C.I., Muñoz M., Buonocore G.G., Aprea P., Liguori B., Verdolotti L. (2020). Fabrication of Green Diatomite/Chitosan-Based Hybrid Foams with Dye Sorption Capacity. Materials.

[103] Novais R.M., Pullar R.C., Labrincha J.A. (2020). Geopolymer foams: An overview of recent advancements. Prog. Mater. Sci..

[104] Gong P.J., Taniguchi T., Ohshima M. (2014). Nanoporous structure of the cell walls of polycarbonate foams. J. Mater. Sci..

[105] Smith R.M. (2002). Extractions with superheated water. J. Chromatogr. A.

