# Supplementary material for: Open-Celled Foams from Polyethersulfone/Poly(Ethylene Glycol) Blends Using Foam Extrusion
Source: Polymers (Basel). 2022 Dec 27;15(1):118. doi: 10.3390/polym15010118 (PMC9824152; doi:10.3390/polym15010118)
Supplement: Supplementary file 1 [file polymers-15-00118-s001.zip › polymers-2045095-supplementary.docx]

Supplementary Materials

**Open-Celled Foams from Polyethersulfone/Poly(Ethylene
Glycol) Blends Using Foam Extrusion**

**Aniket Raje ^1^, Prokopios Georgopanos ^1,^*, Joachim Koll ^1^, Jelena Lillepärg ^1^, Ulrich A. Handge ^1,2^ and Volker Abetz ^1,3^**

^1^ Helmholtz-Zentrum Hereon, Institute of Membrane Research, Max-Planck-Strasse 1, 21502 Geesthacht, Germany; aniket.raje@hereon.de (A.R.); joachim.koll@hereon.de (J.K.); jelena.lillepaerg@hereon.de (J.L.); ulrich.handge@tu-dortmund.de (U.A.H.); volker.abetz@hereon.de (V.A.)

^2^ Chair of Plastics Technology, Faculty of Mechanical Engineering, TU Dortmund University,
Leonhard-Euler-Straße 5, 44227 Dortmund, Germany

^3^ Institute of Physical Chemistry, Universität Hamburg, Grindelallee 117, 20146 Hamburg, Germany

***** Correspondence: prokopios.georgopanos@hereon.de; Tel.: +49-4152-87-2420

Supporting Information





**Figure S1.** Gel permeation chromatography of PESU E 3010 and E 3020 P. The molecular weight estimation is based on calibration with polystyrene standards.

|  |  |
| --- | --- |
| (**a**) | (**b**) |
|  |  |
| (**c**) |  |

**Figure S2.** Rheological investigations on the PESU / PEG200 blends: Storage modulus G’ and loss modulus G’’ versus angular frequency ω at various temperatures of blend (**a**) E3_PEG200_08 (**b**) E3_PEG200_14; (**c**) E3_PEG200_26


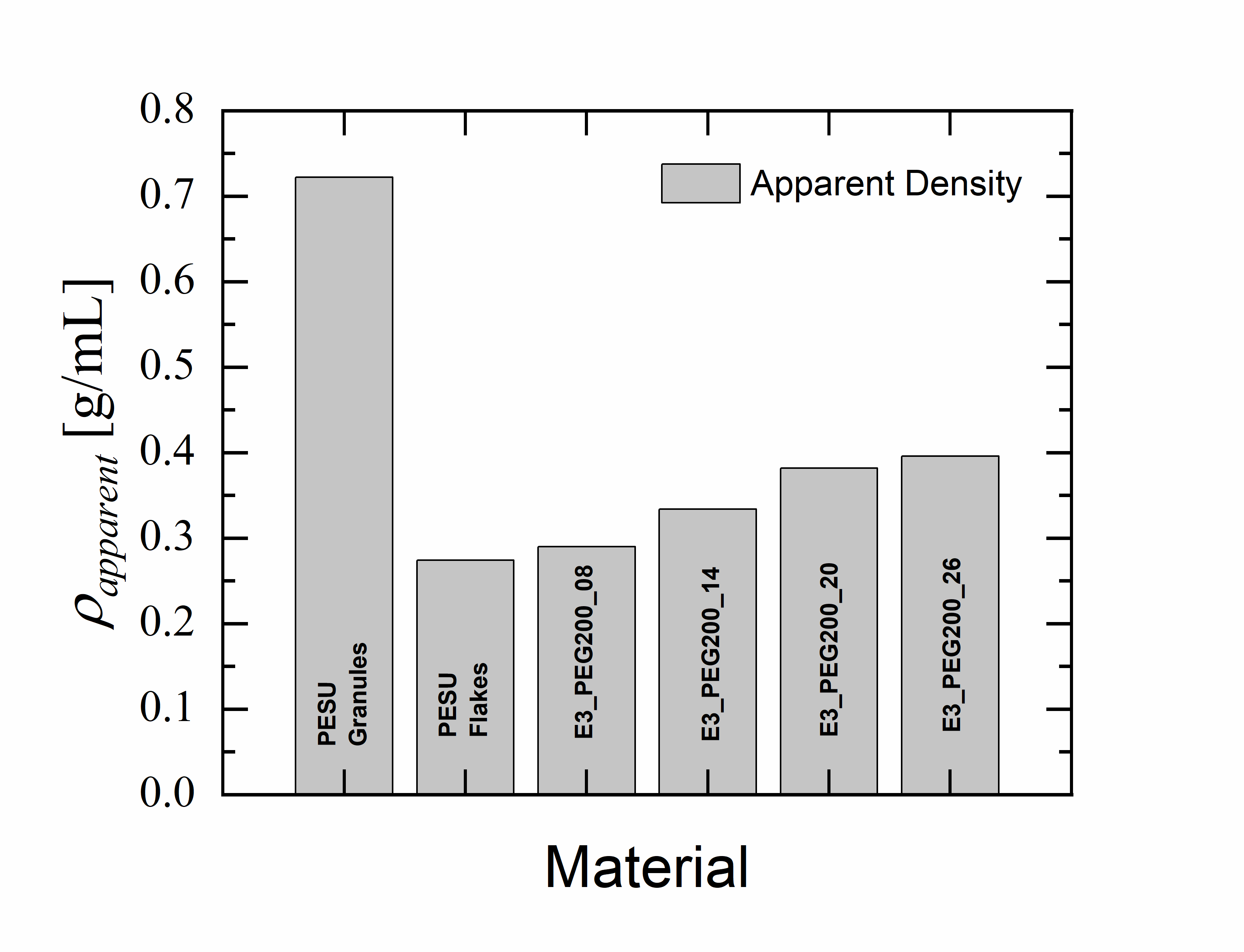


**Figure S3.** Apparent densities of PESU E 3010 granules, PESU E 3020 P flakes and PESU/PEO blend flakes.





**Figure S4.** DSC third heating cycle of foam of blend E3_PEG200_20, extrusion foamed using CO_2_ and water.
